# A magnetic dial for exciton-polaritons

**DOI:** 10.1038/s41377-026-02445-9

**Published:** 2026-08-21

**Authors:** Konstantinos S. Daskalakis

**Affiliations:** Department of Mechanical and Materials Engineering, University of Turku, Turku, Finland

**Keywords:** Optics and photonics, Optical materials and structures

## Abstract

Experiments in the van der Waals magnet CrSBr show that magnetic fields can strongly tune exciton-polariton coupling strength and optical nonlinearity. These results establish magnetic order as a powerful control knob for reconfigurable polaritonic matter.

Few moments in science are as striking as the observation of quantum effects on a macroscopic scale. An important part of this progress has been the ability to study collective quantum phenomena in solid-state platforms, and increasingly at temperatures far above liquid helium. Exciton-polaritons—hybrid light–matter states formed when a material’s electronic excitations, namely excitons, strongly couple to confined electromagnetic modes so that their identities become inseparable—have been among the leading systems in this pursuit. Their light-like component gives them a small effective mass and long-range coherence, while their matter-like component provides interactions and optical nonlinearity. This combination has enabled studies of Bose–Einstein condensation, superfluidity, nonlinear switching, while also offering routes to lowering lasing thresholds, topological photonics and emerging optical-computing concepts^1–5^.

Yet in nearly all these directions, one ingredient remains essential: control. In most polariton systems, the essential parameters are largely fixed during fabrication by the cavity design, material oscillator strength and exciton–photon detuning. Optical pumping, temperature, strain and electrical gating can add tunability, but often with limited range or additional loss. The matter part of a polariton suggests a more direct possibility. If the exciton can be controlled, the polariton should follow. Magnetic semiconductors offer a particularly elegant possibility: by rearranging spin order, an applied magnetic field can modify the exciton and thereby tune the polariton. The rapid development of van der Waals magnets makes this route especially timely^6^. In CrSBr, excitons are already known to interact strongly with magnons^7^, while spin-correlated polaritons in NiPS₃ and self-hybridized polaritons in CrSBr have established magnetic van der Waals materials as a promising polaritonic platform^8,9^.

It is here where the recent work of Li et al. makes an important step forward^10^. The authors study CrSBr, a layered van der Waals antiferromagnetic semiconductor, and focus on a higher-energy exciton near 1.8 eV. This choice is crucial. Earlier CrSBr polariton studies mainly explored the lower-energy exciton near 1.37 eV, whose coupling strength is only weakly affected by magnetic field. The higher-energy exciton, by contrast, has a larger oscillator strength and a wavefunction that is much more sensitive to interlayer spin order.

The experimental platform is also simple. Exfoliated CrSBr flakes act as their own Fabry–Pérot cavities, so that photons trapped in the crystal hybridize with excitons without the need for a separately fabricated microcavity. To substantiate the strong-coupling claim, Li et al. use angle-resolved reflectance, the standard experimental tool for resolving polariton dispersion and anticrossing. From this analysis, they extract Rabi splittings of several hundred meV, including about 632 meV at 6 K and 745 meV at room temperature for a representative flake. Most strikingly, when an in-plane magnetic field drives CrSBr from antiferromagnetic order through an intermediate magnetic phase toward ferromagnetic order, the inferred Rabi splitting decreases by nearly 100 meV within only a few tenths of a tesla. In simple terms, the magnet turns a dial and the polariton splitting changes.

This is more than a large tuning effect. It shows that the coupling strength itself, usually treated as a fixed property of a cavity-material system, can become a dynamic variable controlled by spin order. The reason is microscopic: the higher-energy exciton is spatially more delocalized and therefore more sensitive to field-induced changes in interlayer electronic hybridization. The lower-energy exciton, in contrast, is much less magnetically tunable.

The work also shows that magnetic order can reshape polariton nonlinearity. In the intermediate magnetic phase, where spin alignment is partially disrupted, the nonlinear response is enhanced and can even change sign depending on the polariton branch. Li et al. attribute this behavior to magnon-assisted long-range interactions and magnetic-field-dependent reduction of the coupling strength. This interpretation is supported by transient reflectance measurements showing oscillations near the magnon frequency, but further field-resolved experiments will be important to quantify the mechanism.

Looking ahead, the next challenge is to understand how far this magnetic control can be generalized. The strength of the present platform lies in its simplicity: it gives direct access to spin-sensitive polaritons without elaborate cavity fabrication, but it also ties the optical resonances to the crystal thickness and the strongest response to a particular exciton energy. More broadly, the work highlights that the excitonic component of a polariton is more than a source of interactions and nonlinearity: it is a gateway through which material properties enter photonics. In CrSBr, that gateway carries magnetic order, allowing spin configuration to tune both coupling strength and nonlinear response. Future studies could place CrSBr inside designed cavities, gratings or photonic-crystal structures, where the optical mode can be chosen independently of the crystal thickness. This would make it possible to test which aspects of the response come from the magnetic exciton itself, and which come from the surrounding optical environment. In this view, Li et al. pave the way toward magnetic polaritonic platforms in which spin order becomes an active design parameter for controlling light–matter coupling and nonlinear response (Fig. 1).

**Fig. 1:**
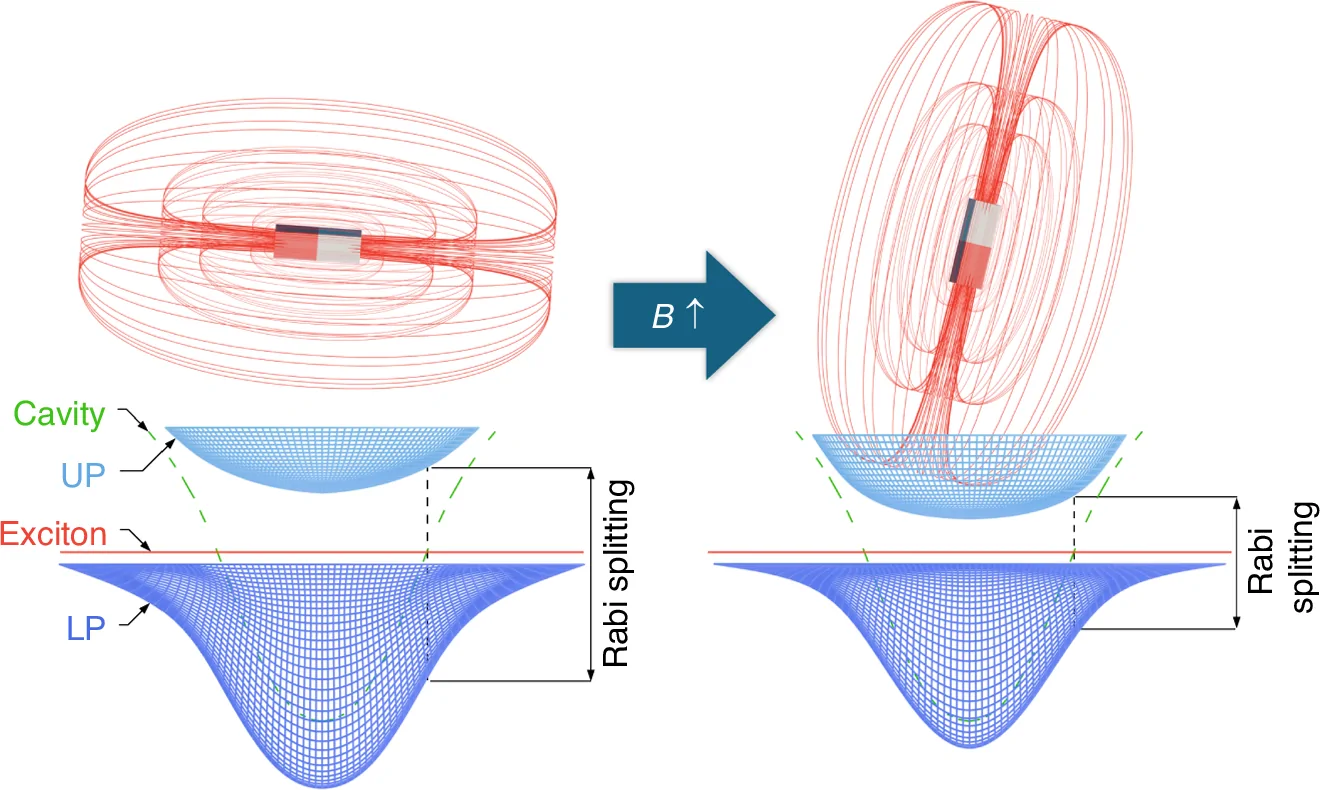
Magnetic-field (B) control of exciton-polariton coupling in CrSBr. A magnetic field changes the spin order of CrSBr, reducing the inferred Rabi splitting in a coupled-oscillator picture. The green dashed curves indicate the uncoupled cavity mode (Cavity), the red line the higher-energy exciton (Exciton), and the blue curves the upper polariton (UP) and lower polariton (LP) modes
